# Comparative effectiveness of surgery versus radiotherapy for non-nasopharyngeal head and neck lymphoepithelial carcinoma: An IPTW propensity score analysis

**DOI:** 10.1371/journal.pone.0321318

**Published:** 2025-04-29

**Authors:** Zhen Zheng, Jiaying Wu, Bing Cao, Yanping Bei, Hui Zhang, Kaitai Liu

**Affiliations:** 1 Department of Chemoradiation Oncology, The Affiliated Lihuili Hospital of Ningbo University, Ningbo, Zhejiang Province, China; 2 School of Nursing, Ningbo College of Health Sciences, Ningbo, Zhejiang Province, China; 3 Department of Otolaryngology Head and Neck Surgery, The Affiliated Lihuili Hospital of Ningbo University, Ningbo, Zhejiang Province, China; West China Hospital of Sichuan University, CHINA

## Abstract

**Background:**

Lymphoepithelial carcinoma (LEC) is a rare head and neck malignancy predominately treated with radiotherapy or surgery. However, comparative effectiveness studies, are lacking for non-nasopharyngeal head and neck LEC (HNLEC).

**Methods:**

Patients diagnosed with non-nasopharyngeal HNLEC from 2000–2019 were extracted from the Surveillance, Epidemiology, and End Results (SEER) database. Inverse probability of treatment weighting (IPTW) was used to balance baseline characteristics. Cancer-specific survival (CSS) was compared between surgery and radiotherapy groups using Kaplan-Meier analyses and Cox regression before and after IPTW adjustment.

**Results:**

248 patients were included. Before IPTW adjustment, treatment modalities were not significantly associated with CSS in both Kaplan-Meier analysis(p=0.065) and univariate Cox regression(p=0.068). After weighting, Kaplan-Meier analysis revealed a significant CSS difference favoring surgery (p=0.015), and univariate Cox regression showed surgery (p=0.018), race (p<0.001), tumor size (p=0.024) and radiotherapy (p=0.0003) as independent predictors. On subgroup analysis of patients receiving single-modality therapy, landmark analysis beyond 60 months showed improved CSS with surgery versus radiotherapy (p<0.001) after IPTW adjustment. Additionally, Kaplan-Meier analysis showed no pre-IPTW (p=0.68) or post-IPTW (p=0.30) CSS differences between surgery alone and surgery plus radiotherapy.

**Conclusion:**

This population-based analysis demonstrated a potential survival advantage of surgery over radiotherapy for non-nasopharyngeal head and neck LEC after accounting for confounding factors. Additional comparative effectiveness data, ideally from controlled studies, are warranted to further investigate optimal treatment strategies.

## Introduction

Lymphoepithelial carcinoma (LEC) is a rare and distinct malignant epithelial neoplasm. Histologically, it is characterized by undifferentiated or poorly differentiated carcinoma cells admixed with prominent lymphoid stroma, similar to undifferentiated nasopharyngeal carcinoma, and is often associated with Epstein-Barr virus (EBV) infection [1,2]. LEC occurs more commonly in men than women, predominantly between 50–70 years of age [1,3–6]. Although LEC predominantly originates in the head and neck region, this malignancy has also been reported in other anatomical sites, including the breast, lung, and stomach [7–10].

For head and neck LEC (HNLEC), the nasopharynx is the most common site, comprising up to 40% of nasopharyngeal neoplasms [11]. Given the anatomical proximity and biological similarities, research on nasopharyngeal carcinoma provides the most insights on HNLEC. Due to its anatomical location, unique biological behavior, and high radiosensitivity, radiotherapy plays a central role in the treatment of nasopharyngeal carcinoma [12]. For residual or recurrent diseases, the standard of care consists of either reirradiation or salvage surgical resection. However, most published data on HNLEC are limited to case reports and small case series [13–17]. While some studies have suggested radical radiotherapy or combined modality therapy with surgery and postoperative radiation for locoregional non-nasopharyngeal HNLEC [15,18], no consensus exists on the optimal treatment approach. Non-nasopharyngeal HNLEC in the Caucasian population remains largely understudied. Although recent analyses using the Surveillance, Epidemiology, and End Results (SEER) database and real-world data have shed some light on the epidemiology of LEC [7,19], the inherent limitations of the databases have resulted in imbalanced baseline characteristics between treatment groups. Thus, the reliability of overall survival analysis using conventional Kaplan-Meier estimation remains questionable. Furthermore, comparative effectiveness studies focusing on primary treatment modalities, particularly surgery versus radiotherapy, are lacking. In this study, we utilized the SEER database in a population-based study and employed inverse probability of treatment weighting (IPTW) to balance baseline characteristics between treatment groups. We also conducted subgroup landmark analyses to investigate the comparative effectiveness of surgery versus radiotherapy for non-nasopharyngeal HNLEC.

## Materials and methods

### Data source

**Fig 1:**
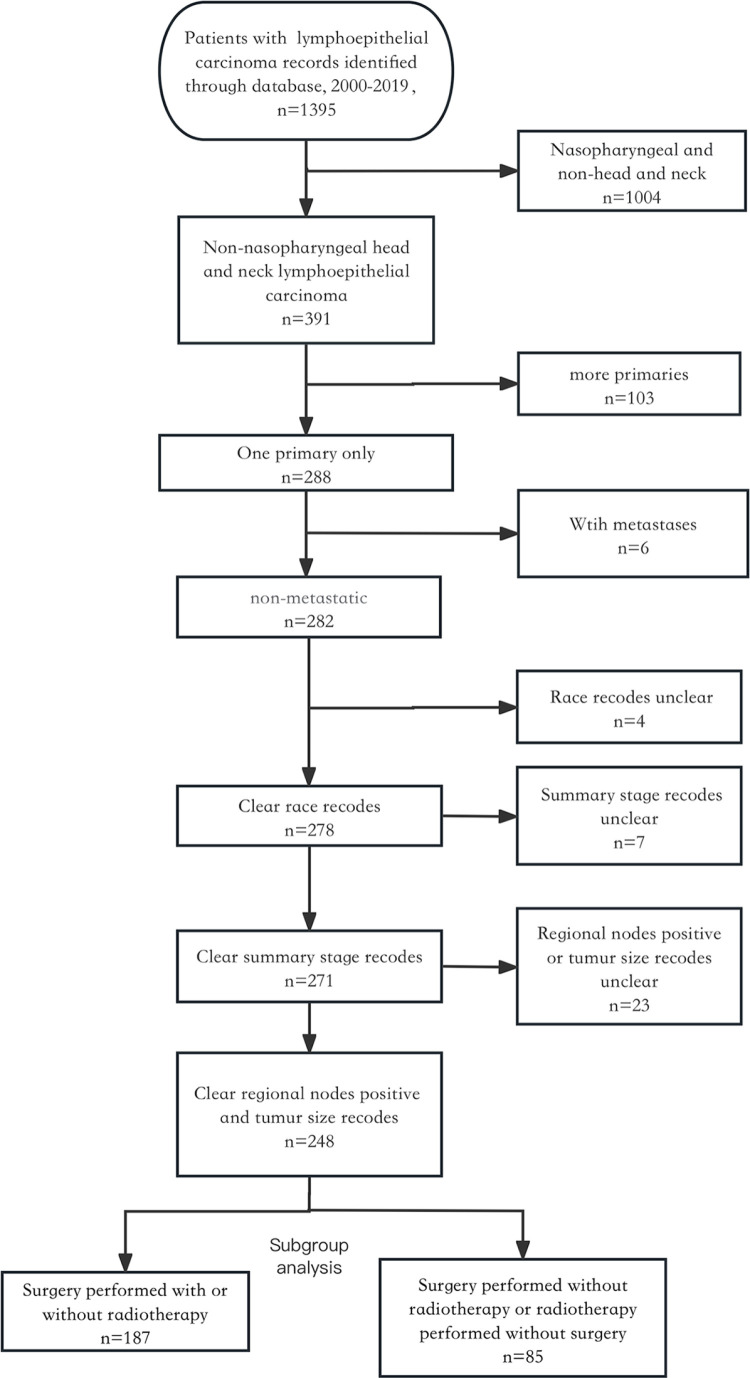
Patient selection flowchart from the SEER database.

This retrospective analysis utilized data from the SEER database (https://seer.cancer.gov/), specifically the SEER*Stat database, Incidence-SEER Research Plus Data, 17 Registries, Nov 2021 Sub (2000–2019). This observational study was reviewed by the affiliated Lihuili Hospital of Ningbo University Research Ethics Committee, which confirmed that no ethical approval was required. Access to the database was granted through official SEER program authorization, and data were accessed on July 7, 2024. Throughout the study period, no access to personal identifiers or potentially identifying information was available, as all SEER data were completely de-identified. The study methodology adhered to the Declaration of Helsinki guidelines. This study was exempt from institutional review board approval as it analyzed de-identified, population-based data from a public database, according to our institutional policies regarding secondary data analysis. The inclusion criteria were as follows: (1) Histology code 8082/3 based on the International Classification of Diseases for Oncology, 3rd Edition (ICD-O-3); (2) Primary tumor site in the parathyroid gland (C75.0); and (3) First primary malignancy only for patients with multiple primaries. Variables extracted included: patient demographics (age at diagnosis, sex, race), tumor characteristics (size, regional lymph node status, SEER summary stage), treatment (radiotherapy, surgery, chemotherapy), survival time, and vital status. Exclusion criteria included: (1) Unknown or <1 month of follow-up; (2) Unknown surgical procedure; (3) Distant metastasis at diagnosis; or (4) Incomplete information. (Fig 1).

### Covariates

Data were extracted on sociodemographic, tumor-related, and treatment characteristics. Sociodemographic variables included age at diagnosis, sex, and race (white, other). Treatment data comprised surgery, radiotherapy, and chemotherapy. Tumor size was categorized by the overall median length of 35 mm. Tumor stage was classified as local, regional, or distant based on SEER codes. The primary outcome was cancer-specific survival (CSS), defined as the interval from diagnosis to cancer-specific death.

### Statistical analysis

Differences in sociodemographic, tumor-related, and treatment-related characteristics between patients treated with surgery alone and surgery plus radiotherapy were assessed using Pearson’s chi-squared test and Fisher’s exact test. IPTW was utilized to balance potential confounding factors influencing surgery or radiotherapy allocation [20]. Balance of baseline characteristics after IPTW was evaluated using the standardized mean difference (SMD). All variables were analyzed using unadjusted and IPTW-adjusted univariate Cox regression analysis. Variables with p <0.05 were included in subsequent analyses. Multivariable Cox regression models were constructed by adjusting for variables selected from the univariate analyses. Adjusted hazard ratios (HR) and 95% confidence intervals (CI) were computed. Both unadjusted and IPTW-adjusted Kaplan-Meier curves were generated and compared via log-rank tests [21]. Subgroup landmark analysis stratified by treatment type was performed with IPTW-adjusted Kaplan-Meier curves and log-rank tests. Landmark analysis at fixed time points was used to reduce immortal time bias [22].

All statistical analyses and plots were performed using R (version 4.3.2). Two-sided p-values <0.05 were considered statistically significant.

## Results

### Characteristics of study subjects

**Fig 2:**
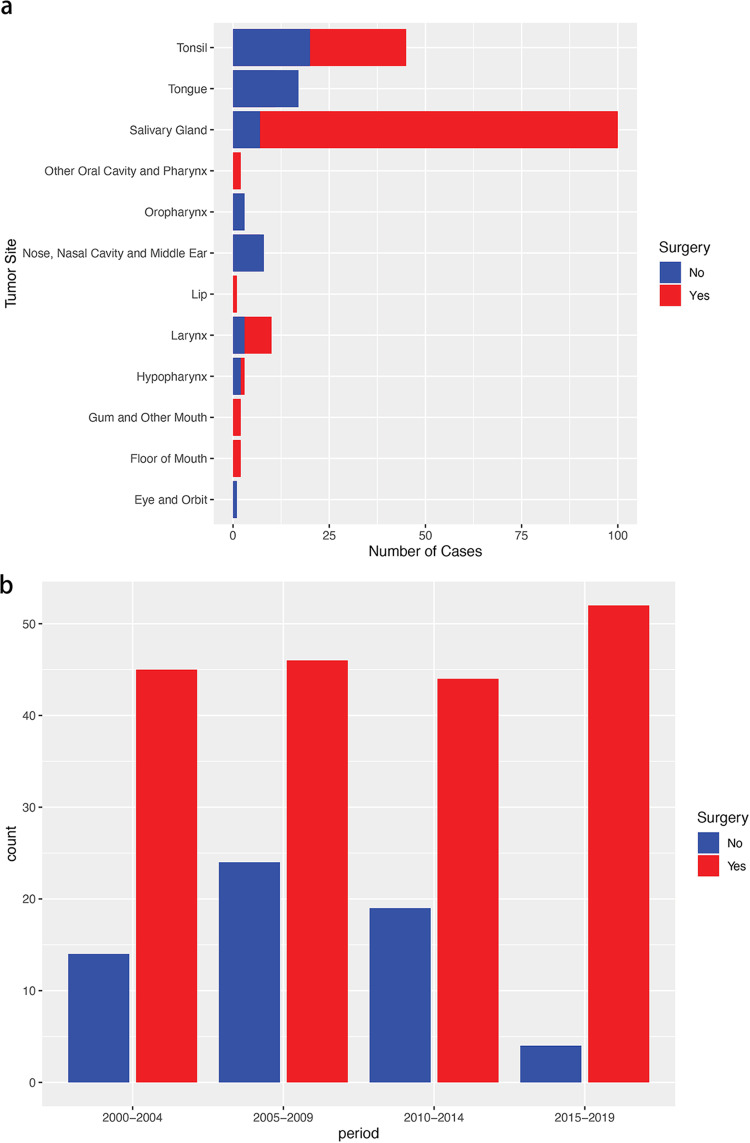
Bar diagram illustrating (a) the sites for patients with non-nasopharyngeal HNLEC; (b) over different diagnosis year in an original unmatched cohort from the SEER database.

A total of 248 patients diagnosed with non-nasopharyngeal HNLEC met the predefined inclusion criteria, with a median follow-up duration of 74.5 months (range: 1–240 months). Table 1 presents the baseline characteristics of the study cohort. Of these patients, 187 underwent surgical resection, and 61 did not receive surgical treatment. The analysis identified 65 years as the cutoff value for age at diagnosis, and 35 mm as the cutoff value for tumor size. The patient demographic profile revealed that 64.9% were male, 69.0% were below the age of 65, 31% were aged 65 or older, and 71.4% were of White race. Additionally, 68.1% of patients had a tumor size less than 35 mm, and 66.1% had positive regional nodes. Radiotherapy and chemotherapy were administered to 83.1% and 42.7% of patients, respectively. Based on the anatomical distribution, Salivary Gland, Tonsil, and Tongue were the top three predominant sites in non-nasopharyngeal HNLEC, with the lowest incidence observed in the Lip (Fig 2a). Analysis of 2000–2019 data showed an increasing trend in surgical management, from 76.3% (45/59) in 2000–2004 to 92.9% (52/56) in 2015–2019. The number of non-surgical cases decreased from 14 patients in 2000–2004–4 patients in 2015–2019, indicating a shift towards surgical intervention (Fig 2b).

**Table 1:** Baseline demographic and clinical characteristics of patients with non-nasopharyngeal HNLEC.

Group	Level	Unmatched			IPTW		
		No Surgery	Surgery	p	No Surgery	Surgery	p
n		61	187		252.5	247.4	
Race (%)	Other	10 (16.4)	61 (32.6)	0.023	73.8 (29.2)	71.1 (28.7)	0.962
	White	51 (83.6)	126 (67.4)		178.7 (70.8)	176.3 (71.3)	
Sex (%)	Female	20 (32.8)	67 (35.8)	0.781	76.6 (30.3)	85.5 (34.6)	0.603
	Male	41 (67.2)	120 (64.2)		175.9 (69.7)	161.9 (65.4)	
Tumor size (%)	<35mm	44 (72.1)	125 (66.8)	0.541	163.5 (64.8)	167.5 (67.7)	0.734
	≥35mm	17 (27.9)	62 (33.2)		89.0 (35.2)	80.0 (32.3)	
Node positive (%)	Negative	13 (21.3)	71 (38.0)	0.026	93.9 (37.2)	84.1 (34.0)	0.738
	Positive	48 (78.7)	116 (62.0)		158.6 (62.8)	163.3 (66.0)	
Age (%)	<65	46 (75.4)	125 (66.8)	0.273	162.5 (64.3)	169.8 (68.6)	0.624
	≥65	15 (24.6)	62 (33.2)		90.0 (35.7)	77.7 (31.4)	
Stage (%)	Localized	6 (9.8)	43 (23.0)	0.040	56.8 (22.5)	49.2 (19.9)	0.775
	Regional+Dista nt	55 (90.2)	144 (77.0)		195.7 (77.5)	198.3 (80.1)	
Radiation (%)	No/unknown	9 (14.8)	33 (17.6)	0.744	48.5 (19.2)	41.7 (16.8)	0.741
	Yes	52 (85.2)	154 (82.4)		204.0 (80.8)	205.8 (83.2)	
Chemotherapy (%)	No/unknown	20 (32.8)	122 (65.2)	<0.001	146.9 (58.2)	142.0 (57.4)	0.927
	Yes	41 (67.2)	65 (34.8)		105.6 (41.8)	105.4 (42.6)	

HNLEC:Non-nasopharyngeal Head and Neck Lymphoepithelial Carcinoma, IPTW:Inverse probability of treatment weighting.

**Fig 3:**
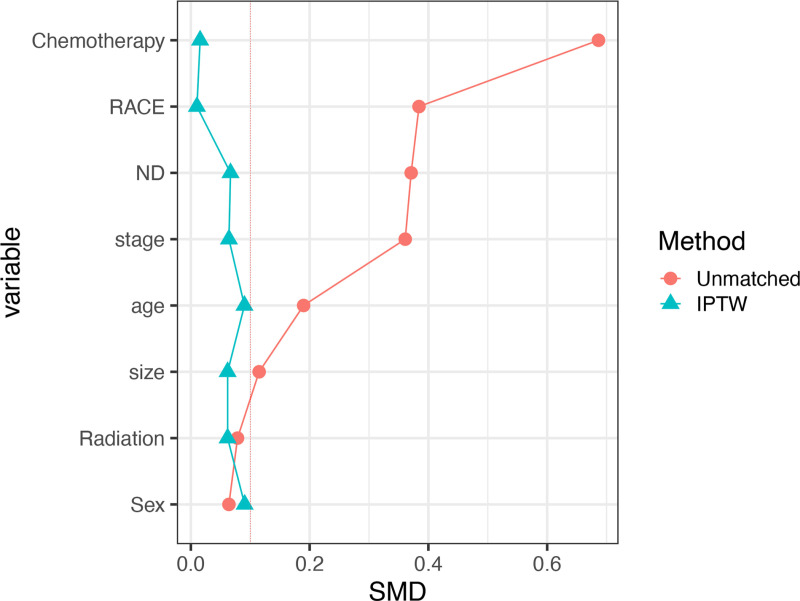
Standardized mean differences of different cohorts.

Before IPTW, SMDs for age at diagnosis, race, tumor size, regional nodes status, chemotherapy, and radiotherapy exceeded 0.1, indicating potential bias in the original data. However, after IPTW adjustment, the SMDs for all variables decreased to less than 0.1, demonstrating comparability between the surgery and no-surgery groups (Fig 3).

### Survival analyses

**Fig 4:**
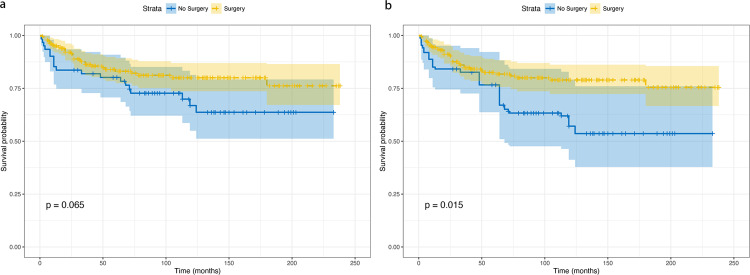
Cancer-specific survival analysis comparing surgical versus non-surgical treatment: (a) unadjusted cohort; (b) adjusted cohort. Median follow-up: 74.5 months; maximum follow-up: 240 months.

The Kaplan–Meier analysis initially indicated no statistically significant difference in CSS of patients with non-nasopharyngeal HNLEC between the surgery and no-surgery groups before IPTW adjustment (p=0.065) (Fig 4a). In the univariate Cox proportional hazards regression model without IPTW, there was no discernible difference in CSS between the surgery and no-surgery groups (p=0.068). The multivariate Cox proportional hazards regression model using raw data confirmed that age (p=0.009) and tumor size (p=0.007) independently served as prognostic factors for CSS in patients with non-nasopharyngeal HNLEC (Table 2).

**Table 2:** Univariable and Multivariable cox regression model for cancer-specific survival in patients with non-nasopharyngeal HNLEC in the unadjusted and adjusted cohort.

Group	Level	Unmatched				IPTW			
		Univariable		Multivariable		Univariable		Multivariable	
		HR [95%CI]	p	HR [95%CI]	p	HR [95%CI]	p	HR [95%CI]	p
Race	Other	Reference	0.672			Reference	**<0.001**	Reference	**0.004**
	White	0.88[0.48–1.61]				0.29[0.16–0.53]		0.34[0.16–0.71]	
Sex	Female	Reference	0.396			Reference	0.15		
	Male	1.30[0.71–2.38]				1.77[0.82–3.83]			
Tumor size	<35mm	Reference	**0.011**	Reference	**0.013**	Reference	**0.024**	Reference	0.093
	≥35mm	2.06[1.18–3.60]		2.03[1.16–3.54]		2.33[1.12–4.84]		1.68[0.92–3.07]	
Node positive	Negative	Reference	0.097			Reference	0.8		
	Positive	1.76[0.90–3.44]				0.89[0.38–2.11]			
Age	<65	Reference	**0.014**	Reference	**0.003**	Reference	0.13		
	≥65	2.04[1.16–3.59]		2.03[1.34–4.17]		1.8[0.85–3.84]			
Stage	Localized	Reference	**0.051**	Reference	**0.032**	Reference	0.712		
	Regional+Distant	2.77[1.00–7.71]		3.09[1.10–8.65]		1.29[0.33–5.00]			
Radiation	No/unknown	Reference	0.066			Reference	**0.003**	Reference	**0.015**
	Yes	0.54[0.28–1.04]				0.32[0.16–0.68]		0.43[0.22–0.85]	
Chemotherapy	No/unknown	Reference	0.162			Reference	0.961		
	Yes	1.49[0.85–2.59]				0.98[0.49–1.96]			
Surgery	No	Reference	0.068			Reference	**0.018**	Reference	**0.005**
	Yes	0.59[0.33–1.04]				0.49[0.27–0.88]		0.42[0.23–0.77]	

HNLEC:Non-nasopharyngeal Head and Neck Lymphoepithelial Carcinoma, IPTW:Inverse probability of treatment weighting.

After IPTW adjustment, Kaplan–Meier analysis revealed a significant difference in CSS between surgery and no-surgery groups (p=0.015) (Fig 4b). The univariate Cox proportional hazards regression model indicated that surgery achieved statistical significance (p = 0.018), while race (p < 0.001), tumor size (p = 0.024), and radiotherapy (p = 0.0003) were identified as independent prognostic factors in the adjusted data (Table 2). In the multivariate Cox proportional hazards regression model incorporating IPTW, surgery (HR 0.42, 95% CI 0.31–2.79, p = 0.005), radiotherapy (HR 0.43, 95% CI 0.35–2.43, p = 0.015), and race (HR 0.34, 95% CI 0.38–2.85, p = 0.004) retained their status as independent prognostic factors for CSS in patients with non-nasopharyngeal HNLEC (Table 2).

### Subgroup analyses

**Fig 5:**
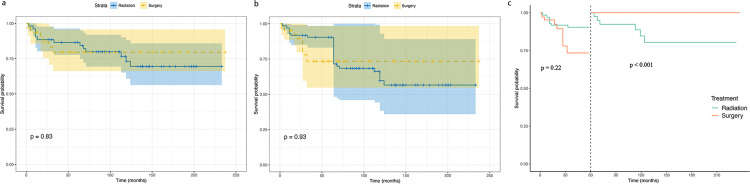
Cancer-specific survival analysis comparing surgery versus radiation therapy: (a) unadjusted cohort; (b) adjusted cohort; (c) landmark analysis of adjusted cohort. Median follow-up: 74.5 months; maximum follow-up: 240 months.

Following the weighted multivariate Cox analysis, which identified both surgery and radiotherapy as independent prognostic factors, we conducted subgroup analysis. Patients were stratified into two groups: surgery without radiotherapy and radiotherapy without surgery, to compare the therapeutic efficacy of these approaches. S1 Table presents the baseline characteristics of each treatment group. Kaplan–Meier analysis showed no significant difference in CSS between these groups both pre-IPTW (p=0.83) (Fig 5a) and post-IPTW adjustment (p=0.93) (Fig 5b). To address potential immortal time bias, landmark analysis was performed at 60 months. Beyond 60 months, the surgery group demonstrated superior CSS compared to the radiotherapy group (p<0.001). However, at the 60-month timepoint, CSS did not differ significantly between the two groups (p=0.22) (Fig 5c).

**Fig 6:**
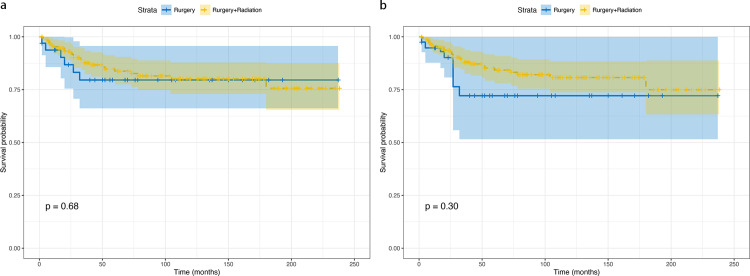
Cancer-specific survival analysis comparing surgery alone versus surgery with radiation: (a) unadjusted cohort; (b) adjusted cohort. Median follow-up: 74.5 months; maximum follow-up: 240 months.

To evaluate the effectiveness of surgery alone versus combined surgery with radiotherapy, a separate analysis was conducted among surgical patients. S2 Table presents the baseline characteristics of the cohort based on the decision to undergo surgery. Kaplan–Meier analysis showed no significant difference in CSS between surgery-only and surgery-with-radiotherapy groups, both pre-IPTW (p=0.68) and post-IPTW adjustment (p=0.30) (Fig 6).

## Disscusion

To our knowledge, this study represents the first attempt to conduct IPTW and landmark analysis in non-nasopharyngeal HNLEC. The IPTW methodology has been successfully applied in other rare cancers to reduce selection bias and balance confounding factors. For example, Qiu et al. utilized IPTW analysis to evaluate treatment outcomes in soft tissue sarcoma with lymph node metastasis, demonstrating its value in comparing different therapeutic approaches when randomized trials are not feasible [23]. Similar to their methodological approach, our study employed IPTW to minimize potential selection bias between treatment groups. Our key findings demonstrated that after IPTW adjustment, the Kaplan-Meier analysis revealed patients undergoing surgery achieved significantly improved cancer-specific survival compared to non-surgical treatment. Results from both univariate and multivariate Cox analyses established surgery as a strong independent predictor of improved CSS, along with radiotherapy and race as significant prognostic factors. Notably, our landmark analysis at 60 months revealed that surgical intervention conferred superior long-term survival benefits compared to radiotherapy. This finding was further supported by our temporal trend analysis showing an increasing preference for surgical management from 76.3% in 2000–2004 to 92.9% in 2015–2019, suggesting a shift in treatment paradigm towards surgical intervention.

LEC is an uncommon malignancy characterized by undifferentiated, non-keratinizing squamous cell carcinoma associated with prominent lymphocytic infiltration. It bears morphological similarity to lymphoepithelioma first described in the nasopharynx by Regaud and Schmincke in 1921, as noted by Ewing [24]. Although rare in general, LECs tend to arise in various head and neck sites. Previous studies have implicated viral infections in the pathogenesis of HNLEC [15,25-27]. High EBV prevalence has been reported in studies from endemic regions such as Taiwan, Hong Kong, Russia and Greenland [25,26,28,29]. In contrast, human papillomavirus (HPV) infection appears more prevalent in western HNLEC cohorts [27,30] The largest analysis by Singhi et al. found all 19 evaluated oropharyngeal LELC cases in the United States to be positive for HPV-16 but negative for EBV [27] Our multivariate analysis confirmed race (p=0.004) as an independent prognostic factor after applying IPTW，which may be related to differences in viral infection among different race.

As LECs lack a capsule and have a strong tendency for early metastasis, over 40% of patients present with cervical lymph node involvement. Around 20% develop local recurrence or nodal spread, while another 20% experience distant metastases within 3 years despite therapy [31–34]. Reported 5-year survival ranges from 70% to 91% [28,35]. Previous studies using the SEER database have identified race, gender, age, and treatment disparities impacting LEC survival [7,36,37]. However, the prognostic significance of these factors for HNLEC remains unclear, as prior analyses have included nasopharyngeal carcinoma patients. In 2015, Jason Y. K. Chan et al. analyzed demographic, clinicopathologic and survival data for HNLEC cases in the SEER database. Older age, surgery omission and lack of radiation were independently associated with worse survival outcomes [7]. The optimal treatment strategy for non-nasopharyngeal HNLEC remains controversial. Currently accepted modalities include surgery and/or radiotherapy, with or without chemotherapy. Recent studies have explored different treatment approaches with varying results. Niu et al. found that stereotactic radiotherapy (SRT) provided better progression-free survival compared to surgery alone in salivary gland LELC, though overall survival differences were not significant [38]. In contrast, an earlier study by Dubey et al. reported poor outcomes with radiotherapy alone, showing 5-year regional control and overall survival rates of 83% and 39% respectively [18]. The role of chemotherapy in improving survival remains unclear. Our study did not find an association between chemotherapy and increased cause-specific survival, which aligns with findings by Ma et al. showing no survival benefits from postoperative chemotherapy in primary salivary gland diseases [15]. A real-world study from Southern China examined outcomes of different treatment approaches and found that compared to definitive radiotherapy ± chemotherapy, surgery alone was associated with significantly higher 3-year local (96.5% vs 92.8%, p=0.012) and regional (96.8% vs 89.3%, p=0.002) relapse-free survival, while definitive radiotherapy and SRT showed comparable 3-year survival rates [19]. Compared to definitive radiotherapy +/- chemotherapy (SRT), surgery alone was associated with significantly higher 3-year local (92.8% vs 96.5%, p=0.012) and regional (89.3% vs 96.8%, p=0.002) relapse-free survival. Definitive radiotherapy and SRT demonstrated comparable 3-year survival rates (all p>0.05). However, to our knowledge, no studies have performed weighted analysis to account for baseline confounding or directly compared surgery versus radiotherapy for HNLEC. Moreover, data directly comparing treatment outcomes for non-nasopharyngeal HNLEC are scarce. Our study innovatively utilizes IPTW and landmark analysis to investigate treatment effectiveness, suggesting surgery may confer survival benefit.

Several studies have aimed to identify valuable biomarkers that can offer therapeutic insights to enhance the outcomes of patients with HNLEC [39,40]. Joshua Sckolnick and colleagues examined 19 cases of HNLEC in the United States to investigate evidence of microsatellite instability at the DNA level and alterations in the DNA mismatch repair (MMR) system at the immunohistochemical staining level. Ultimately, they found that alterations in the DNA MMR system are not a common mechanism of tumorigenesis in HNLELC of the head and neck in a nonendemic country [40]. Another study led by Wen-Qing Zou found that PD-L1, B7H3, and IDO-1 were predominantly expressed in the tumor nest. A checkpoint-based signature emerged as an independent predictor, significantly enhancing the predictive performance of TNM stage for 3-year (p = 0.014), 5-year (p = 0.056), and 10-year disease-free survival (p = 0.023) [39]. The mechanism behind lymphocyte infiltration in EBV-positive tumors requires further investigation. While the elimination of EBV-positive malignant cells may trigger cytotoxic lymphocyte recruitment, the precise immunological pathways involved remain to be elucidated.

Several important limitations of this study should be acknowledged. First, as with all registry database analyses, the SEER database lacks crucial information about treatment quality, including radiation doses, field dimensions, specific chemotherapy regimens, and detailed surgical procedures. Second, important potential confounders such as patient performance status, comorbidities, smoking history, and socioeconomic factors are not recorded, which limited our ability to perform comprehensive risk stratification analyses. Third, the absence of recurrence data and treatment-related toxicity information prevents analysis of disease-free survival, patterns of failure, and treatment complications. These limitations necessitate cautious interpretation of our results and highlight the need for prospective studies with more detailed clinical information to definitively guide treatment selection.

## Conclusion

In conclusion, our weighted analysis of real-world data demonstrates that surgical treatment significantly improved survival outcomes compared to non-surgical approaches in non-metastatic cases, with an increasing trend toward surgical management observed over the past two decades. These findings suggest surgery may confer an effectiveness advantage over radiotherapy, though they should be interpreted within the context of registry database limitations. Additional high-quality evidence from prospective multi-institutional observational studies is warranted to clarify the comparative roles of surgery and radiotherapy in the definitive management of locoregional disease.

## Supporting information

S1 Table Baseline characteristics of the distinct treatment cohorts within the non-nasopharyngeal HNLEC population. (XLSX)

S2 Table Baseline characteristics of the cohort based on the decision to undergo surgery. (XLSX)
